# Supplementary material for: Transcriptome analyses reveal differences in the response to temperature in Florida and Northern largemouth bass (Micropterus spp.) during early life stages
Source: PLoS One. 2025 Feb 18;20(2):e0317563. doi: 10.1371/journal.pone.0317563 (PMC11835285; doi:10.1371/journal.pone.0317563)

***Supplementary Material for:***

**Transcriptome analyses reveal differences in the response to temperature in Florida and Northern largemouth bass (*Micropterus spp.*) during early life stages**

Authors: Moisés A. Bernal, Gavin L. Aguilar, Josh Sakmar, Sebastian N. Politis, Savannah L. Oglesby, Allen Nicholls, Anita M. Kelly, Luke A. Roy, Ian A.E. Butts

**Supplementary Table 1.** Total length, body area and standard deviation for Florida and North, based on 100 random individuals. Data modified from Aguilar et al. (2023).

|  | DPH | Temp | Total Length | SD | Body area | SD | N |
| --- | --- | --- | --- | --- | --- | --- | --- |
| Florida | 8 | 21 | 7.71 | 0.06 | 5.65 | 0.14 | 100 |
|  | 8 | 24 | 8.196 | 0.05 | 6.92 | 0.26 | 100 |
|  | 8 | 27 | 8.72 | 0.12 | 7.4 | 0.12 | 100 |
|  | 28 | 21 | 12.04 | 0.08 | 25.57 | 0.311 | 100 |
|  | 28 | 24 | 15.89 | 0.5 | 30.23 | 2.35 | 100 |
|  | 28 | 27 | 19.2 | 0.63 | 46.07 | 2.44 | 100 |
| NorthERN | 8 | 21 | 7.31 | 0.01 | 5.61 | 0.03 | 100 |
|  | 8 | 24 | 7.79 | 0.011 | 6.703 | 0.03 | 100 |
|  | 8 | 27 | 8.169 | 1.58 | 8.64 | 0.06 | 100 |
|  | 28 | 21 | 12.56 | 0.044 | 28.53 | 0.37 | 100 |
|  | 28 | 24 | 16.9 | 0.024 | 42.46 | 0.295 | 100 |
|  | 28 | 27 | 19.92 | 0.05 | 51.78 | 0.072 | 100 |

******S Fig1. WGCNA dendrogram clustering based on gene composition dissimilarity of the detected modules.** Results for Florida (A) and North (B) lineages.

B.

A.

**S Fig 2. Correlation between module membership and gene significance for the most significant modules at 27°C.** Results for Florida (Green module - A) and North (Brown module -B) lineages.


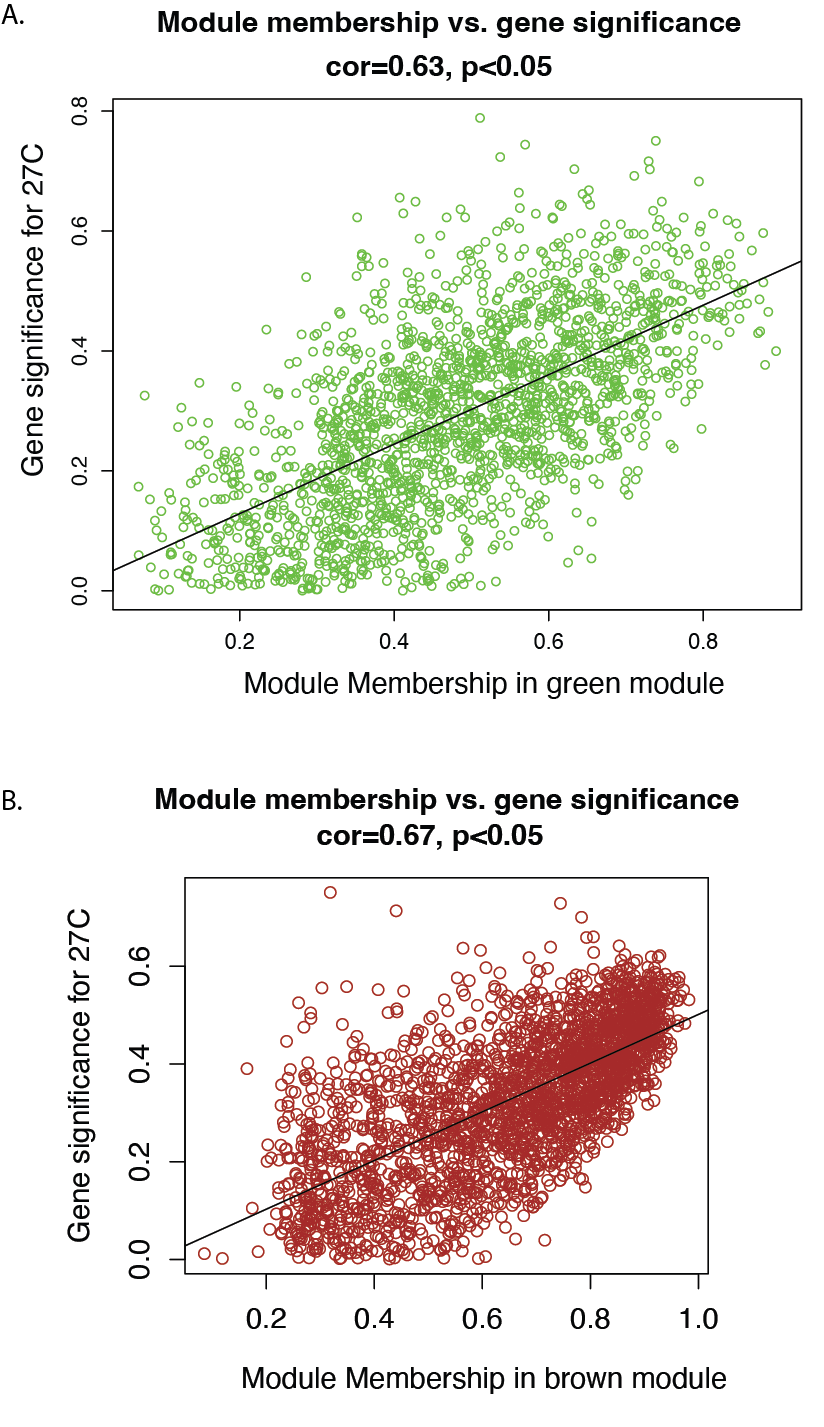

Supplement: S1 File — (DOCX) [file pone.0317563.s001.docx]
