# Supplementary material for: Transcriptome analyses reveal differences in the response to temperature in Florida and Northern largemouth bass (Micropterus spp.) during early life stages
Source: PLoS One. 2025 Feb 18;20(2):e0317563. doi: 10.1371/journal.pone.0317563 (PMC11835285; doi:10.1371/journal.pone.0317563)
Supplement: Attachment — Submitted filename: Reviewer report.docx [file pone.0317563.s002.docx]

In general, this is an interesting and well-organized manuscript. The authors sequenced and annotated the transcriptome from two different species of largemouth bass larvae in responding to divergent temperatures. The resulting differential expression profiles reveal some genetic aspects that reflect changes of temperature at early development stages of LMBs. Here, I listed some questions relevant to experimental design and data analysis. If the authors can adequately address these questions in the revised manuscript, I will suggest to accepting the revision for publication.

1. According to the preliminary studies (line 81 - 82), 27 ^o^C can be seeing as the optimal growing temperature for LMBs and their range of tolerating is quite large. So, my question is what’s the reason that three experimental temperatures (21, 24 and 27 ^o^C) were selected? Why is that only colder (21 and 24 ^o^C) temperatures were examined as control (21 ^o^C) and intermediate (24 ^o^C) in the current study? Do you have plan to evaluate warmer living conditions (>28 ^o^C) for LMBs?
2. The authors defined critical early life stages as <30-days post hatch (line 98), and therefore, larvae at 8-DPH and 28-DPH were selected to be examined in the present study. Do you have any literature evidence to support this notion that <30-DPH is significant to larval development and/or critical to aquacultural husbandry, which considering as justifying the experimental design?
3. Is there any specific reason for the authors to use Trinity to assembling transcriptomic sequences (line 200)? Since Trinity is known to have problems handling lowly expressed transcripts and high coverage regions, and its normalization process is also questionable (Honaas et al., 2016) and (<https://github.com/trinityrnaseq/trinityrnaseq/wiki/Trinity-FAQ>).
4. Although the authors summarized the experimental conditions with fish treatments in Table 1, there is no detail information stating how many samples were sequenced and how the technical and biological repeats were generated. Please provide this information for further evaluation of the reliability of experiment. In addition, do you pool tissue? If not, how do you generalize the bias between individuals?
5. In regarding to determining the differential expression genes (DEGs), since there are two variables included in the experimental design (temperatures and age: DPH), I will need the authors to clarify how to normalize the sequencing data into relative abundances of each mRNA in representing relative expression levels of each gene?
6. The qualities of picture and diagram in Figure 2, 3, and 5 are poor. Please revise these figures with better quality.
7. For Figure 5, What does the color bar indicate to? Does the red color indicating the degree of positively correlated and blue color indicating the degree of negatively correlated to certain GO terms? Clarification and a more meaningful figure legend are required.
