# Supplementary material for: Transcriptome analyses reveal differences in the response to temperature in Florida and Northern largemouth bass (Micropterus spp.) during early life stages
Source: PLoS One. 2025 Feb 18;20(2):e0317563. doi: 10.1371/journal.pone.0317563 (PMC11835285; doi:10.1371/journal.pone.0317563)
Supplement: Attachment — Submitted filename: Review Comments_1131005.docx [file pone.0317563.s003.docx]

The manuscript (PONE-D-24-37286) presents a technically sound study using transcriptomic data to explore how two lineages of largemouth bass respond to temperature variations during early life stages. The data is well-supported by the methodology, and the conclusions regarding differential gene expression under various temperature conditions are valid based on the presented results. The inclusion of gene ontology (GO) and weighted gene co-expression network analysis (WGCNA) enriches the interpretation of the functional significance of differentially expressed genes (DEGs). However, to ensure the completeness of the content, there are some areas that could be improved:

The introduction provides a solid overview of the importance of temperature regulation in early fish development. However, the link between the specific hypotheses tested in this study and previous research on largemouth bass transcriptomics could be made clearer. I recommend adding more detail on how this study builds upon existing work in this field, specifically emphasizing the novelty of comparing the two largemouth bass lineages (Florida and Northern). This addition would provide a stronger foundation for the reader to understand how the study advances the current knowledge on fish transcriptomics and thermal adaptation.

The description of the experimental design could benefit from further elaboration on the triplicate data used for each treatment group. The manuscript currently lacks sufficient detail on the replication strategy for the two largemouth bass lineages (Florida and Northern) across the three temperature conditions. Please clarify how the triplicates were incorporated into the experimental setup and any statistical methods used to account for this in the analysis. This is essential for reproducibility and understanding the robustness of the data.

Page 15: In Table 2, which presents the contig statistics and BUSCO results for the Florida and Northern largemouth bass transcriptomes, there is a notable difference in the "Fragmented BUSCOS" category. Specifically, the Florida largemouth bass transcriptome has 113 fragmented BUSCOs (3.1%) compared to 83 fragmented BUSCOs (2.3%) in the Northern transcriptome. This discrepancy warrants further discussion. Could this difference be due to variations in sequencing quality or depth between the two lineages, or might it reflect underlying biological factors such as genetic divergence between the populations? I recommend addressing this point in the manuscript, perhaps by providing additional details on the assembly process or discussing potential biological explanations. Clarifying this difference would enhance the readers' understanding of the assemblies' quality and their implications for downstream analyses.

Page 17: Figure 4. There appears to be an inconsistency between Figure 4 and its caption. The caption refers to comparisons across temperature treatments at different developmental stages for both Florida (A) and Northern (B) largemouth bass, but it also implies the existence of panels (C) and (D), which are either missing or mislabelled. To ensure clarity and accurate representation of the data, I suggest revising the figure to include the missing panels or adjusting the caption to match the figure as currently presented. Consistent labeling of figures and captions is essential for clear communication of the results.

The presentation style of Table 3 resembles more of a diagram than a traditional table, which may lead to confusion. For better clarity and consistency with the rest of the manuscript, I recommend reformatting Table 3 into a standard table layout with clear rows and columns. This will make the data more accessible and easier to interpret for the reader, thereby improving the overall presentation of the results.

Figure 5 is well-structured, but there are areas where the caption and its reference in the manuscript can be improved. Specifically:

1) Please use consistent terminology for "Weighted Gene Network Correlation Analysis" (WGCNA). The abbreviation WGCA is also mentioned elsewhere—ensure uniformity throughout the manuscript.
2) Clarify how correlation strength and statistical significance are represented in the figure. The description of the correlation coefficients and p-values could be more transparent for readers unfamiliar with the methodology.
3) Ensure that the manuscript provides a thorough biological interpretation of the correlations shown in Figure 5, particularly highlighting key findings and their implications for understanding gene expression in response to temperature across developmental stages.

The discussion section interprets the findings effectively within the broader context of thermal tolerance in fish. However, it would benefit from a deeper exploration of how these transcriptomic changes might affect long-term survival, growth, and reproduction in the wild. Additionally, there are opportunities to discuss the potential applications of these findings in management, conservation, and aquaculture. For instance, how might selective breeding programs leverage these results to improve thermal tolerance in largemouth bass populations? Expanding on these points could provide greater relevance to the study's broader ecological and practical implications.
